# New perspective on the regionalization of the anterior forebrain in *Osteichthyes*

**DOI:** 10.1111/dgd.12348

**Published:** 2017-05-04

**Authors:** Kei Yamamoto, Solal Bloch, Philippe Vernier

**Affiliations:** ^1^ Paris‐Saclay Institute of Neuroscience (UMR 9197) CNRS Université Paris‐Sud Université Paris‐Saclay Gif‐sur‐Yvette 91190 France

**Keywords:** evolution, hypothalamus, optic recess region, telencephalon, ventricle

## Abstract

In the current model, the most anterior part of the forebrain (secondary prosencephalon) is subdivided into the telencephalon dorsally and the hypothalamus ventrally. Our recent study identified a new morphogenetic unit named the optic recess region (ORR) between the telencephalon and the hypothalamus. This modification of the forebrain regionalization based on the ventricular organization resolved some previously unexplained inconsistency about regional identification in different vertebrate groups. The ventricular‐based comparison also revealed a large diversity within the subregions (notably in the hypothalamus and telencephalon) among different vertebrate groups. In tetrapods there is only one hypothalamic recess, while in teleosts there are two recesses. Most notably, the mammalian and teleost hypothalami are two extreme cases: the former has lost the cerebrospinal fluid‐contacting (CSF‐c) neurons, while the latter has increased them. Thus, one to one homology of hypothalamic subregions in mammals and teleosts requires careful verification. In the telencephalon, different developmental processes between *Sarcopterygii* (lobe‐finned fish) and *Actinopterygii* (ray‐finned fish) have already been described: the evagination and the eversion. Although pallial homology has been long discussed based on the assumption that the medial‐lateral organization of the pallium in *Actinopterygii* is inverted from that in *Sarcopterygii*, recent developmental data contradict this assumption. Current models of the brain organization are largely based on a mammalian‐centric point of view, but our comparative analyses shed new light on the brain organization of *Osteichthyes*.

## Graphical abstract

We propose a new perspective on the regionalization of the vertebrate forebrain. Our morphological analyses focusing on the ventricular organization challenge current hypotheses of regional homology of the telencephalon and hypothalamus.

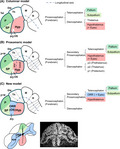

## Introduction

**Figure 1:**
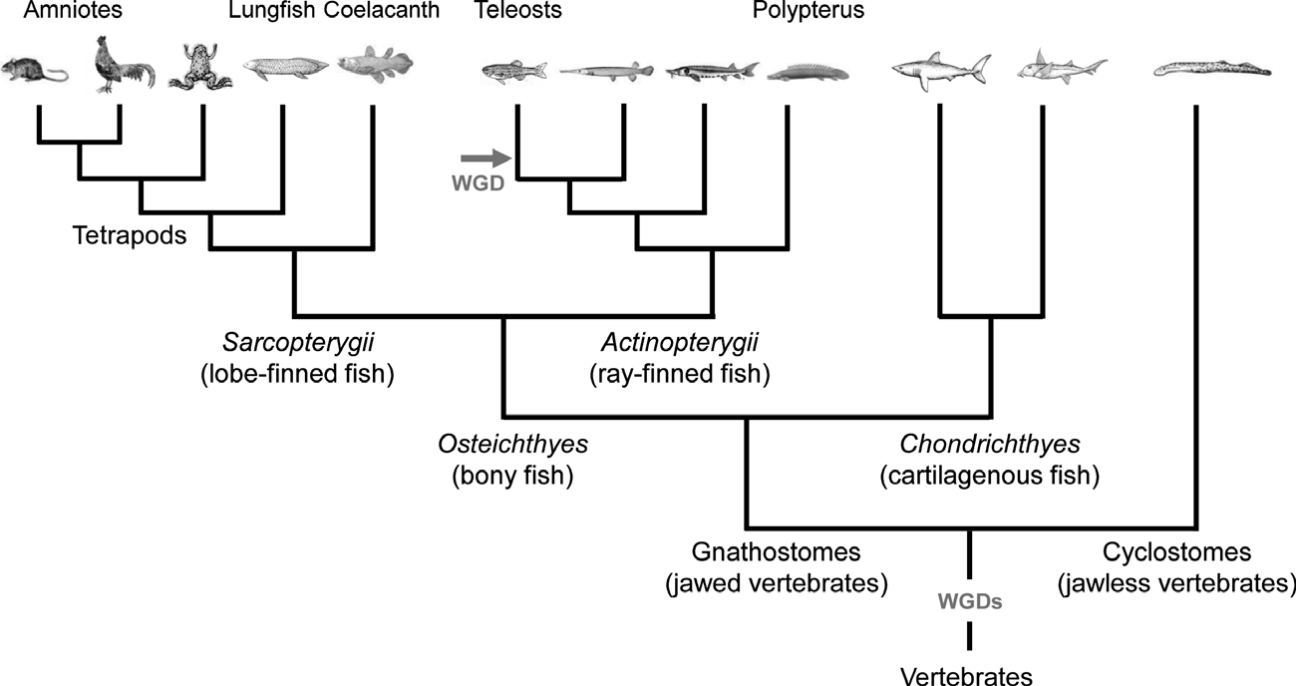
Phylogenetic tree of vertebrates. A simplified phylogenetic tree focusing on the evolution of *Osteichthyes* (bony fish). *Osteichthyes* is divided into two categories: *Sarcopterygii* (lobe‐finned fish) that contains tetrapods, and *Actinopterygii* (ray‐finned fish) that contains teleosts. Based on recent findings, it is hypothesized that two rounds of whole genome duplication (WGD) occurred before the gnathostomes‐cyclostomes split. The teleost lineage went through an additional WGD.

The living vertebrates are divided into gnathostomes (jawed vertebrates) and cyclostomes. Cyclostomes are the only group of living agnathans (jawless vertebrates), and they consist of much fewer species (such as the lamprey and hagfish, ±110 species) than living gnathostomes (±54 650 species). The gnathostomes are divided into two large groups, *Osteichthyes* (bony fish, ±53 700 species) and *Chondrichthyes* (cartilaginous fish, ±970 species). The group of *Osteichthyes* contains *Sarcopterygii* (lobe‐finned fish, ±26 750 species) and *Actinopterygii* (ray‐finned fish, ±27 000 species; see Bally‐Cuif & Vernier 2010). Note that so‐called “fishes” such as lungfish or coelacanths are phylogenetically closer to tetrapods than to ray‐finned fishes. Indeed, the tetrapod is a group of lobe‐finned “fish” specialized for terrestrial life (Fig. 1).

Although the overall Bauplan (plan of construction) of the brain is conserved, significant differences exist within each brain region in different vertebrate groups. Due to the close phylogenetic relationship to humans, a majority of studies have been performed in mammalian species such as rat and mouse, and thus current views of the brain organization largely rely on the mammalian data. However, developmental and comparative studies in non‐mammalian vertebrates give new perspectives on the general organization of the brains.

In this review, we focus on the forebrain organization of *Osteichthyes*, because most of the data come from the comparative analyses of the tetrapod and teleost brains. Many brain features described here are shared throughout the vertebrates, but we will avoid broad generalization since we will not discuss studies from *Chondrichthyes* and cyclostomes.

## Different models of forebrain regionalization: columnar versus neuromeric models

How a simple neural tube develops into an elaborate brain is a major question for neuroanatomists and developmental biologists. The sequential developmental processes of the vertebrate brain integrate several concomitant events: morphogenesis of the neural tube, positional information (antero‐posterior and dorso‐ventral axes) specifying the cell fate, and differentiation producing different cell types in the brain (Edlund & Jessell 1999; Wilson & Houart 2004; Vieira *et al*. 2010).

It has been generally accepted that the neural tube gives rise to three primary morphological domains (or vesicles). These three domains are the forebrain (prosencephalon), the midbrain (mesencephalon), and the hindbrain (rhombencephalon) that is continuous with the spinal cord (the central nervous system is composed of the brain and spinal cord). Although a recent study has suggested that the existence of the initial three vesicles is not universal to all vertebrates (Ishikawa *et al*. 2015), most studies of brain development have been based on this trichotomy of the neural tube.

**Figure 2:**
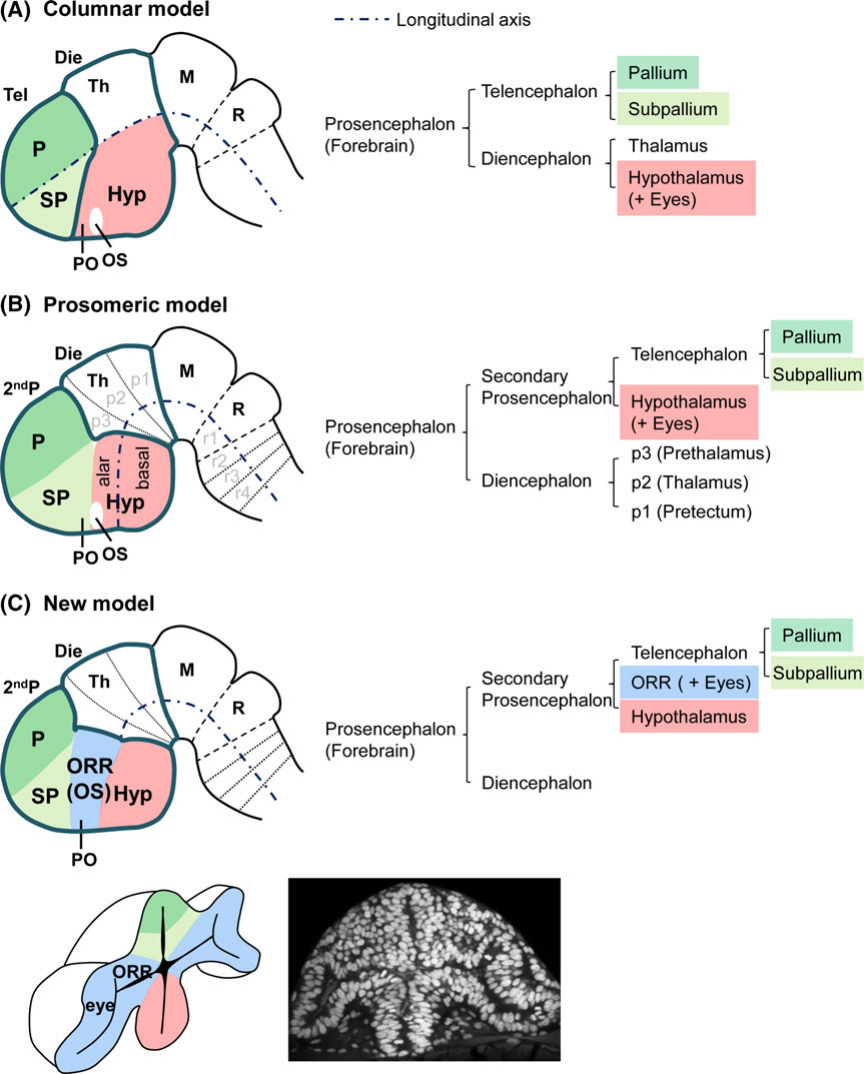
Different models for the subdivision of the forebrain. Representative vertebrate brains from a lateral view (rostral to the left) are shown to demonstrate three different models. (A) The columnar model in which the hypothalamus is considered to be the ventral half of the diencephalon. (B) The prosomeric model which was originally proposed by Puelles and Rubenstein in the early 1990s and has been modified over time. In this model, the hypothalamus is proposed to be the ventral half of the most anterior part of the forebrain, and the telencephalon and hypothalamus consists of the secondary prosencephalon. (C) A new model proposed by Affaticati *et al*. (2015), in which the secondary prosencephalon is divided into three parts, the telencephalon, hypothalamus, and optic recess region (ORR). At the bottom, 3D illustration (left; modified from Picker *et al*. 2009) and a confocal image of DAPI staining (right) of a frontal section of a zebrafish embryo demonstrate that the eyes are continuous with the ORR. 2ndP, secondary prosencephalon; Die, Diencephalon; Hy, hypothalamus; M, mesencephalon; ORR, optic recess region; OS, optic stalk; P, pallium; p, prosomeric subdivision; PO, preoptic area; R, rhombencephalon; r, rhombomeric subdivision; SP, subpallium; Tel, telencephalon; Th, thalamus.

The first three vesicles (forebrain, midbrain, and hindbrain) are further divided into five vesicles. In a classical view, the hindbrain is subdivided into the myelencephalon (containing the medulla oblongata) caudally and the metencephalon (containing the cerebellum and pons) rostrally. The midbrain is considered to remain one division by itself. The forebrain is subdivided into the diencephalon caudally and the telencephalon rostrally. The diencephalon is further divided into the thalamus dorsally and the hypothalamus ventrally, and the telencephalon is further divided into the pallium dorsally and the subpallium ventrally (Fig. 2A columnar model; Herrick 1910; also reviewed in Puelles & Rubenstein 2015).

Based on current developmental biology, the brain regionalization depends on the establishment of subdivisions along the anterior‐posterior (A‐P) and dorso‐ventral (D‐V) axes of the neural tube. The concept of A‐P segmentations was further refined by establishment of the neuromeric model (Fig. 2B). Neuromeres are defined as transversal divisions which appear transiently in the developing neural tube. In the hindbrain, segmentations called rhombomeres are clearly observable and shaped by specific genetic and cellular mechanisms. Each segment is named r1, r2, r3….from rostral to caudal (Keynes & Lumsden 1990; Kiecker & Lumsden 2005). In this model, the cerebellum is a bulge at the roof of r1, and nerve fibers of different cranial nerves (sensory and motor innervation to the face) are organized along the rhombomeres.

The same idea was applied to the forebrain, and the prosomeric model was proposed based on morphological landmarks and gene expression patterns (Fig. 2B; Puelles & Rubenstein 2003). In this view, the forebrain is subdivided into the posterior “diencephalon” whose development is influenced by the notochord, and the anterior “secondary prosencephalon” influenced by the prechordal plate. These “secondary organizers” located in the mesodermic tissue ventral to the neural tube secrete a morphogen sonic hedgehog (Shh). Other secondary organizers such as the *zona limitans intrathalamica* (ZLI) and the anterior neural ridge (ANR) are located within the forebrain, secreting morphogens along the A‐P axis (Vieira *et al*. 2010).

Due to the complex morphology of the forebrain, the prosomeric model has been more controversial, and several modifications have been introduced over time (Puelles & Rubenstein 2003, 2015; Puelles *et al*. 2013). The diencephalon is further divided into three prosomeres termed p1 (pretectum), p2 (thalamus), and p3 (prethalamus), from posterior to anterior. So‐called dorsal thalamus in the adult brain develops from p2, and the ventral thalamus develops from p3. In the amniote brain, the dorsal thalamus is extremely enlarged and occupies the majority of the diencephalon. Due to this enlargement of p2 during development, the anterior p3 is pushed ventrally. The secondary prosencephalon is the anterior end of the A‐P axis and it contains telencephalon dorsally and the hypothalamus ventrally. Although the hypothalamus was originally defined as a region which resides ventral to the thalamus (as the name “hypo”‐thalamus indicates), it is now considered to be the most anterior part of the neural tube (Puelles & Rubenstein 2015). The dorsally located telencephalon contains the pallium and subpallium. In the embryonic brain, the subpallium is anterior (instead of ventral) to the pallium, but in animals possessing a large pallium (such as mammals and birds), expansion of the pallium later pushes the subpallium ventrally.

The prosomeric model was established mostly based on the development of the mouse and chicken brains. Gene expression patterns (mainly transcription factors) are often used to delineate subregions of the brain (genoarchitecture). Since these genes are well conserved in vertebrates, they are convenient to identify homologous brain regions, thus the model was subsequently applied to other vertebrate species. For example, the expression of *Dlx* genes was used as a marker of the subpallium, and the expression of *Otp* was used as a marker of the supraoptoparaventricular region (SPV; a part of the “alar hypothalamus”). These genes were used to delineate the telencephalic/hypothalamic border in the Xenopus brain (Dominguez *et al*. 2013). However, when this model is applied to the teleost brain, borders delineated by the gene expression patterns do not coincide with morphogenetic borders. A recent study analyzing the morphogenesis of the teleost forebrain led to suggest some modifications in the prosomeric model.

## A new model of the regionalization of the secondary prosencephalon

Based on the 3D analysis of proliferation and differentiation markers in the embryonic zebrafish brain, Affaticati *et al*. (2015) propose that the secondary prosencephalon is formed from three distinct embryonic morphogenetic units: the telencephalon, hypothalamus, and newly identified optic recess region (ORR) that is continuous with the retina in the eye (Fig. 2C; Affaticati *et al*. 2015).

**Figure 3:**
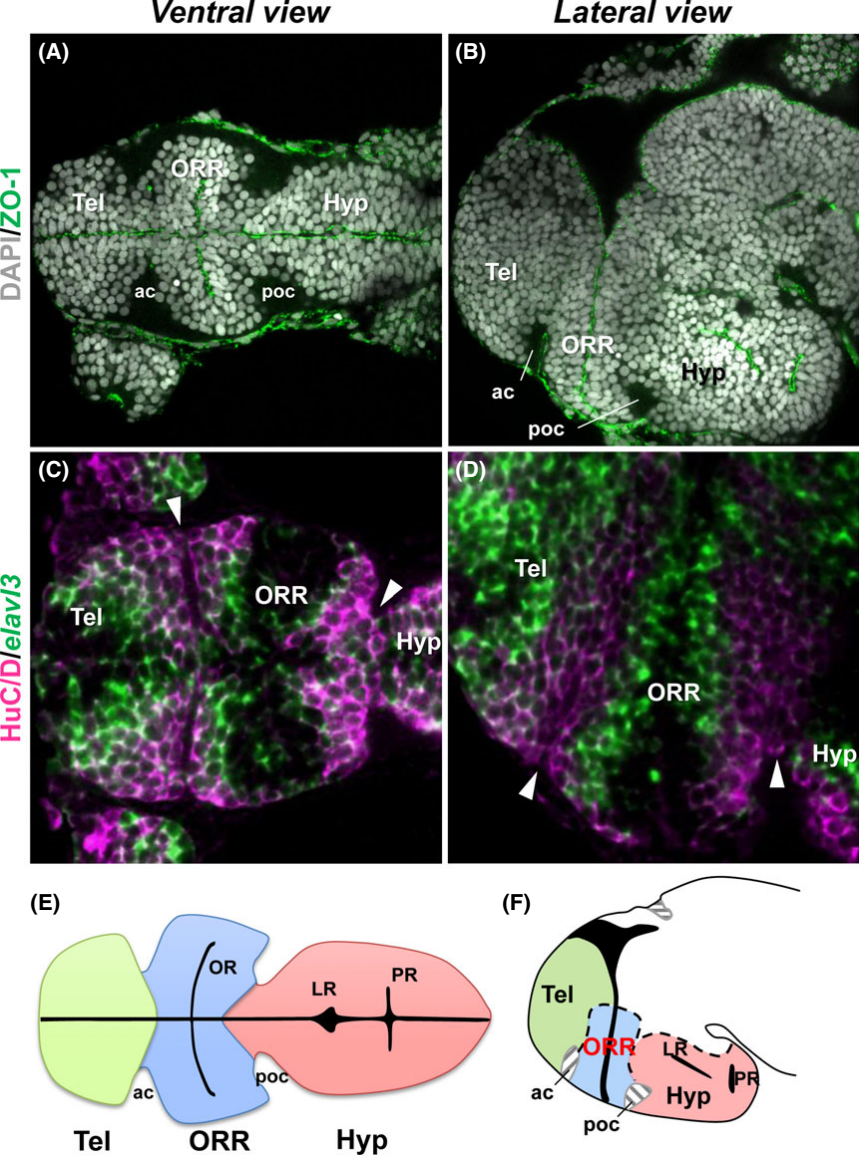
Regional boundaries defined by abutting differentiated neurons. (A, B) Ventral (A) and lateral (B) views of the zebrafish embryonic brain showing three cell masses, the telencephalon (Tel), optic recess region (ORR), and hypothalamus (Hyp). Cell nuclei are labeled with DAPI (gray) and ventricular zones are labeled with ZO‐1 (green). (C, D) Ventral (C) and lateral (D) views of the zebrafish embryonic brain showing gradient cell maturation from the ventricular zones. Differentiating cells are labeled with *elavl3* (green) and differentiated neurons are labeled with HuC/D (magenta). Two rows of HuC/D cells originating from different ventricular zones are in apposition at boundaries (arrowheads) of Tel, ORR, and Hyp. (E, F) Schematic drawing of the ventral (E) and lateral (F) views of the embryonic zebrafish brain, showing the telencephalon (green), ORR (blue), and hypothalamus (red). Simple anatomical landmarks of their boundaries are anterior (ac) and postoptic (poc) commissures. ac, anterior commissure; Hyp, hypothalamus; LR, lateral recess; OR, optic recess; ORR, optic recess region; poc, postoptic commissure; PR, posterior recess; Tel, telencephalon.

The first important point of this new view is that it takes into consideration the morphogenetic process radially organized around the ventricles. The telencephalon, ORR, and hypothalamus can be defined as three distinct brain regions that develop around three ventricular systems: the telencephalic ventricle, optic recess, and hypothalamic ventricle (Fig. 3A,B). Centrifugal organization of neurogenesis from the ventricle to the periphery of the neural tube forms each of the brain regions. At the border between these regions, two rows of differentiated cells (HuC/D immunopositive cells) originating from different ventricular zones are found (Fig. 3C,D). Thus, the regional boundaries can be drawn by the abutting differentiated neurons originated from the different ventricular zones. The anterior and postoptic commissures, which are commonly present in vertebrates (Suárez *et al*. 2014), can be used as simple anatomical landmarks for these boundaries. The anterior commissure (ac) marks the boundary of the telencephalon and ORR, and the postoptic commissure (poc) marks the boundary of the ORR and hypothalamus (Fig. 3A,B,E,F).

This model does not necessarily contradict the neuromeric model in the more caudal part of the brain. In the secondary prosencephalon, however, the simple transversal segmentation does not take into account the evagination of the eyes.

**Figure 4:**
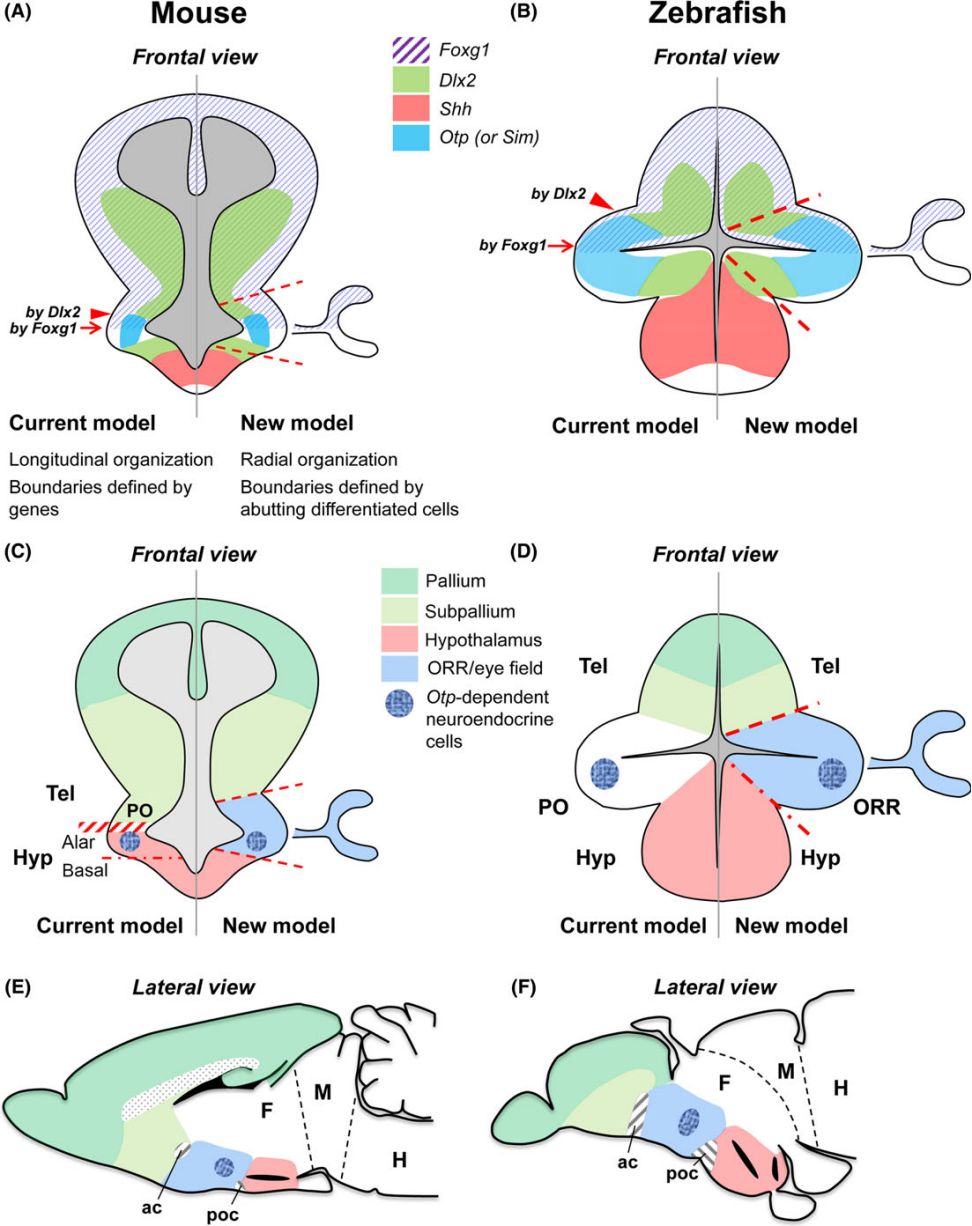
A new model solving discrepancies of homology in the current model. Frontal (A–D) and lateral (E–F) views of the anterior forebrain in mouse (A, C, E) and zebrafish (B, D, F). Frontal planes (A–D) represent embryonic brain sections showing both current (left) and new (right) models on the regional identity of the anterior forebrain. (A, B) The color code represents gene expression data in mouse (A) and zebrafish (B). In the current view (left side of the brain), the ventral limit of the telencephalon is often delineated by the expression of *Dlx2* (arrowhead) or *Foxg1* (arrow), but the two borders do not coincide (which is more prominent in the teleost brain). In the new model, regional boundaries are delineated by abutting differentiated neurons, and they do not necessarily correspond to the limit delineated by gene expression. (C, D) The color code represents proposed regional identity in mouse (C) and zebrafish (D). In the current model established in amniotes (left side of the brain in C), the preoptic area (PO) is considered to be a part of the subpallium due to the expression of F*oxg1* or *Dlx* genes, and *Otp*‐dependent neuroendocrine cells are considered to be located in the hypothalamus. While in teleosts, the area called the PO contains the *otp*‐dependent neuroendocrine cells (left side of the brain in D). In the new model, the corresponding area is the optic recess region (ORR). Considering the *Otp*‐positive area in mouse as the ORR solves the discrepancy of the homology of neuroendocrine cell population between amniotes and teleosts. (E, F) Lateral view of mature brain sections of mouse (E) and zebrafish (F), representing the new model on the regional identity of the anterior forebrain. The color code of each region is the same as C and D. The anterior commissure (ac) and postoptic commissure (poc) can be simple anatomical landmarks for the regional boundary.

Another important point is that the boundaries drawn using a set of transcription factors (*Foxg1*,* Dlxl2*,* Shh*,* Otp*, etc.) that have been used as “regional markers” do not fit the regional boundary defined by the differentiated cells (Fig. 4A,B). Since homologous genes are expressed in a similar pattern between different species, they are useful to identify homologous areas or cell populations. However, the gene expression patterns cannot be used to define the regional boundaries accurately. This leads to a reassessment of the interpretation of the “genoarchitecture” of the developing brains (Puelles & Ferran 2012; Puelles *et al*. 2013).

The identification of the ORR as a morphogenetic unit in addition to the telencephalon and the hypothalamus solves some previously unexplained inconsistency about regional identification in different vertebrate groups. Moreover, the comparative analysis focusing on the ventricular organization helps to better understand morphogenetic processes, and thus provides new perspectives on the homology of brain subdivisions or cell populations. In the rest of this article, we will discuss these points region by region.

## Optic recess region (ORR): a morphogenetic unit organized around the optic recess

The ORR is clearly continuous to the retina of the eye in the teleost embryo (Fig. 2C), and it corresponds to the area that has been identified as the “optic stalk”, which is flanked by anterior and postoptic commissures both in mouse and zebrafish (Shimamura *et al*. 1995; Wilson & Houart 2004). In addition, optic vesicles (which form optic cups) develop around the optic recess in a very similar manner as ORR (Picker *et al*. 2009; Ivanovitch *et al*. 2013). These data suggest that ORR may be a part of the eye field.

The presence of ORR would be a common feature in vertebrates, although in amniotes, the ORR has been difficult to be identified due to its relatively small size compared to the enlarged telencephalon. Comparison of gene expression patterns suggests that the transcription factors we found in the zebrafish ORR (*Foxg1*,* Dlx2*,* Otp*,* Sim*, etc.) are expressed in the telencephalic stalk region in amniotes (Fig. 4A), including the optic stalk (Marcus *et al*. 1999; Roy *et al*. 2013).

The new model of the forebrain regionalization also clarifies the homology of the *Otp*‐dependent neuroendocrine cell population (Affaticati *et al*. 2015). In both amniotes and teleosts, several categories of neuroendocrine cells regulating pituitary functions are located in the territory expressing the transcription factor *Otp*. Based on the neurochemical and gene expression data, they are suggested to be homologous, but in amniotes the area containing these neuroendocrine cells has been identified as the hypothalamus, whereas in teleosts they are located in the preoptic area (PO; Herget *et al*. 2014; Biran *et al*. 2015). In the new framework, these *Otp*‐expressing neuroendocrine cells are located in the ORR, both in amniotes and in teleosts (Fig. 4C–F).

Indeed, the ORR corresponds to the PO of the mature teleost brain. A structure named the PO is also present in tetrapods, but the tetrapod PO does not exactly correspond to the same domain as the teleost PO (Fig. 4C,D). When the new model is applied to the tetrapod brain, the ORR includes domains that have been considered to be the “subpallial PO”, and a part of the alar hypothalamus (Fig. 4C), thus reconciling this previous discrepancy.

## Hypothalamus

The new model of the forebrain regionalization also changes the definition of the hypothalamus in tetrapods. In the prosomeric model, the hypothalamus is divided into the alar and basal parts (Figs 2B, 4C). As mentioned above, a part of the “alar hypothalamus” containing the neuroendocrine cells is now considered to be a part of the ORR (Fig. 4C–F), which leads to the reduction of the “hypothalamus proper” in tetrapods.

**Figure 5:**
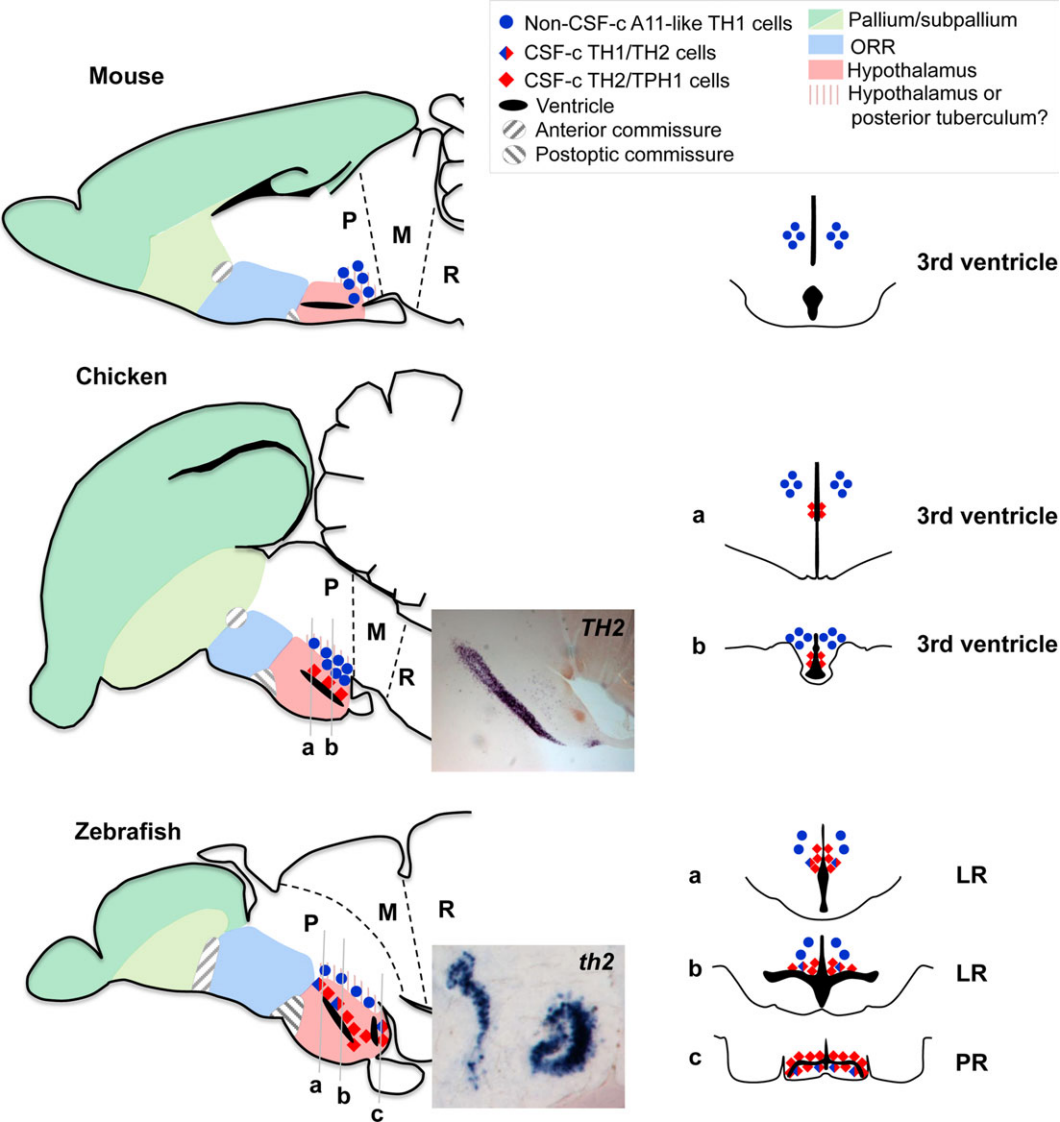
Comparison of the hypothalamic organization in mouse, chicken, and zebrafish. Lateral views of mouse, chicken, and zebrafish brain sections (rostral to the left) are shown on the left, and frontal views around the hypothalamic recess are shown on the right (levels of the frontal planes are indicated with gray lines in the lateral view). In mouse and chicken, there is only one hypothalamic recess (3rd ventricle), while in zebrafish, there are two: the lateral recess (LR) and the posterior recess (PR). Some comparable monoaminergic cell groups are plotted on the schematic drawings. *TH2*/*TPH1*‐expressing CSF‐c DA and 5‐HT cells (red diamonds) are commonly found along the hypothalamic recesses throughout vertebrates (images shown in chicken and zebrafish), while mammals have lost the monoaminergic CSF‐c cells. The A11‐like *TH1*‐expressing DA cell population (blue dots; non CSF‐c cells) projecting to the spinal cord is commonly found dorsolateral to the CSF‐c cells in different vertebrate groups. It is not clear whether the corresponding area is the hypothalamus or the posterior tuberculum (vertical pink lines). LR, lateral recess; M, mesencephalon; ORR, optic recess region; P, prosencephalon; PR, posterior recess; R, rhombencephalon.

Most notably, the hypothalamus is even smaller in placental mammals, since the mammalian hypothalamus lacks cerebrospinal fluid‐contacting (CSF‐c) neurons that are present in the hypothalamic region of all other vertebrates. The CSF‐c cells are located along the periventricular zones and extend processes to contact the CSF. A majority of the hypothalamic CSF‐c neurons contains monoamines, and our recent data demonstrate that they express transcripts of synthetic enzymes of dopamine (DA) and serotonin (5‐HT; Fig. 5; red diamonds; Yamamoto *et al*. 2011; Xavier *et al*. 2017). The presence of the monoaminergic CSF‐c cells in the hypothalami of *Actinopterygii*,* Chondrichthyes* (cartilaginous fishes), and cyclostomes (jawless vertebrates), suggests that they were present in the common ancestor of vertebrates, and have been secondarily lost in placental mammals (Smeets & Reiner 1994; Vígh *et al*. 2004).

In contrast, teleosts possess an enlarged hypothalamus. Due to the large morphological differences, it has been difficult to compare the hypothalamus between teleosts and tetrapods. Taking into consideration the ventricular organization helps to better compare the hypothalamus also. Most vertebrates possess only one hypothalamic recess (around which CSF‐c cells are located), while teleosts possess two hypothalamic recesses: the lateral recess (LR) and the posterior recess (PR; Figs 3E,F, 5 zebrafish). LR is comparable to the tetrapod hypothalamic recess that is a continuation of the third ventricle. PR is only present in some groups of *Actinopterygii* including teleosts and non‐teleost fishes such as gar (Parent & Northcutt 1982) and sturgeon (Kotrschal *et al*. 1985). Since PR is neither found in Polypterus (one of the most basal group of *Actinopterygii*; López & González 2014) nor in *Chondrichthyes* (sister group of *Osteichthyes*; Stuesse *et al*. 1994; see Fig. 1 for the phylogenetic relationship), it is likely that the ancestor of jawed vertebrates did not possess PR. In teleosts, the area surrounding PR is almost exclusively composed of CSF‐c cells, and apparently mammals have no counterpart of the hypothalamic area around the PR. Thus, it is possible that there are some important hypothalamic functions which have never been addressed because they do not exist in mammals.

In teleosts, the PR region has been considered to be the caudal end of the hypothalamus, and the area dorso‐caudal to it has been identified as the posterior tuberculum, a part of the ventral diencephalon. A recent publication by Biran *et al*. (2015) claims that a part of the posterior tuberculum containing A11‐like DA cells (*otp*‐dependent, sending distal efferent to the spinal cord) is indeed a part of the hypothalamus. This is because the A11‐like DA cells in amniotes are located in the area called the mammillary hypothalamic area (Medina *et al*. 1994; Reiner *et al*. 1994; Tillet 1994). As it was the case for the neuroendocrine cells of ORR, the DA cell populations of the posterior tuberculum (which is a part of the diencephalon) and those of the hypothalamus are proposed to be homologous, having a discrepancy in the regional identity between animal groups (Fig. 5; area with vertical pink lines). The proposition by Biran *et al*. (2015) is to modify the regional identity to the one defined in amniotes, yet a boundary between the hypothalamus and the diencephalon remains to be more precisely defined.

An additional difference between teleosts and amniotes is that teleosts do not have an area corresponding to the median eminence of amniotes. In amniotes, the hormones secreted by neuroendocrine cells (majority of which are located in ORR) are released at the median eminence level and transported by the portal blood system to the anterior pituitary, while in teleosts, ORR neuroendocrine cells directly innervate the anterior pituitary (Ball 1981; Fontaine *et al*. 2015).

Due to such large differences in the basic organization, one to one homology of the hypothalamic cell populations among different vertebrate groups requires careful verification. The current model of the hypothalamic organization is largely based on studies of the mammalian hypothalamus that cannot be considered a representative of the vertebrate hypothalamus. To better understand the general organization, more comprehensive analysis needs to be undertaken, including a larger range of vertebrate species.

## Telencephalon

The telencephalon is located at the dorsal part of the anterior end of the neural tube, above the ORR. It is subdivided into the pallium and the subpallium. In the adult telencephalon, the subpallium is located ventral to the pallium, but based on embryological observations, the subpallium is topologically anterior to the pallium (Fig. 2). In this review, we focus on the organization of the pallium, which remains controversial.

**Figure 6:**
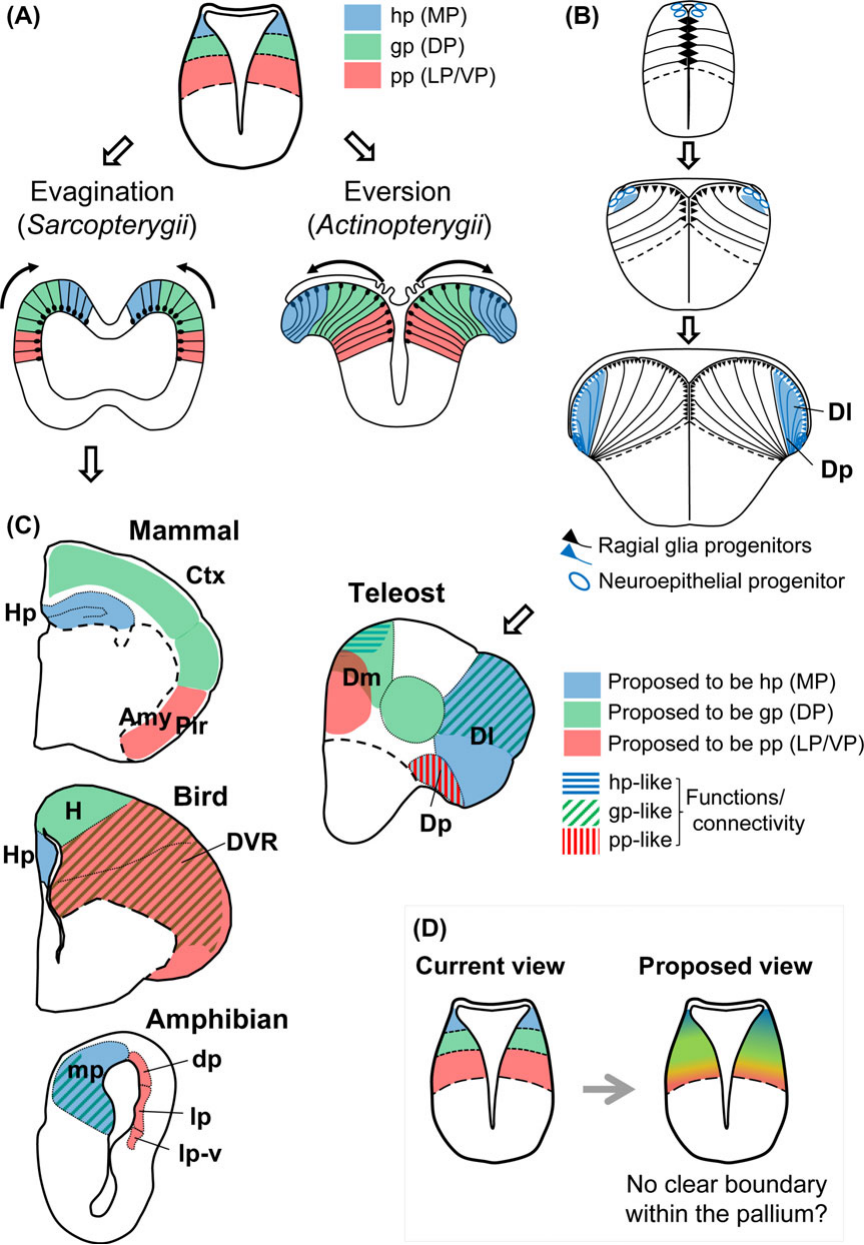
Organization of the pallium in *Osteichthyes*. (A) Schematic drawings showing the classical view of evagination (left) and eversion (right) of the pallium. The color codes represent a proposed homologous pallial region between *Sarcopterygii* and *Actinopterygii* based on the assumption that the pallial organization of *Actinopterygii* is an inverted version of the pallium of *Sarcopterygii*. Based on this theory, the piriform pallium (pp; red) originates from the ventral end of the pallium in both *Sarcopterygii* and *Actinopterygii*. (B) Schematic drawings modified from Dirian *et al*. (2014) showing the pallial development in zebrafish. The lateral and posterior zones of the teleost pallium (Dl and Dp) are formed from the dorsal tip of the pallium, and neurogenesis of this region starts much later than the rest of the pallium. (C) Schematic drawings of frontal sections of mature brains in the mammal, bird, amphibian, and teleost (midline on the left). The colors indicate brain regions proposed to be homologous to the MP (blue), DP (green), and LP (red), respectively. The horizontal blue, slanted green, and vertical red lines indicate pallial areas functionally similar to the hippocampal pallium, general pallium, and piriform pallium, respectively (but the topology does not fit the classical view). (D) Proposed modification of the concept of pallial subdivisions. We propose that there is no distinct subdivision within the pallium. The topology is not a critical factor for determining the pallial properties, and any part of the pallium has potential to generate hippocampal‐like, cortical‐like, piriform‐like, and amygdala‐like characteristics. Amy, amygdala; Ctx, neocortex; Dm, medial zone of dorsal telencephalic area; Dl, lateral zone of dorsal telencephalic area; DP, dorsal pallium (morphotype subdivision); dp, dorsal pallium (amphibian structure); Dp, posterior zone of dorsal telencephalic area; DVR, dorsal ventricular ridge; gp, general pallium; H, hyperpallium; Hp, hippocampus; hp, hippocampal pallium; LP, lateral pallium (morphotype subdivision); lp, lateral pallium (amphibian structure); lp‐v, ventral part of the lateral pallium (amphibian structure); MP, medial pallium (morphotype subdivision); mp, medial pallium (amphibian structure); Pir, piriform cortex; pp, piriform pallium; VP, ventral pallium.

The developmental process of the pallium is largely different between *Sarcopterygii* and *Actinopterygii* (Fig. 6A). In most vertebrates, including *Sarcopterygii*, the telencephalon develops via a process termed evagination. In this process, the central lumen of the neural tube enlarges to form two lateral ventricles (Fig. 6A; left). In *Actinopterygii*, the lateral ventricles are not formed, since the roof of the neural tube elongates outwards to cover the pallium. This way of morphogenesis has been called “eversion” (Fig. 6; right).

According to the classical eversion theory, the medial‐lateral organization of the pallium in *Actinopterygii* is inverted from that in *Sarcopterygii*. Based on this assumption, several hypotheses of the “morphotype” (a model comprising the characteristics believed to have been present in common ancestors [Northcutt 1995]) of the vertebrate pallium have been proposed over the years. However, a recent cell lineage study in zebrafish suggests that the developmental process of the teleost pallium does not go through the “eversion” process exactly as previously believed. Cell lineage tracing demonstrated that the lateral part of the zebrafish pallium containing Dl (lateral zone of dorsal telencephalic area) and Dp (posterior zone of dorsal telencephalic area) originates from a distinct population of progenitors located in the dorsal tip of the embryonic pallium at a later time point than the rest of the pallium (Fig. 6B; Dirian *et al*. 2014). This new data highlights the discrepancy on the classical view of the pallial morphotype, which is explained in more detail below.

Most of the current hypotheses of the pallial homology largely depend on the proposal of Holmgren, which claims three pallial subdivisions: the hippocampal pallium (hp), general pallium (gp), and piriform pallium (pp; Fig. 6; Holmgren 1922, 1925). The hippocampal pallium corresponds to the medial‐most pallial area (that is named the hippocampus in mammals), and general cortex corresponds to the six‐layered cerebral cortex receiving thalamic afferent inputs in mammals. The piriform pallium contains a superficial three‐layered structure (the piriform cortex), and a deep nuclear structure containing the claustrum/amygdala complex (the piriform lobe; Fig. 6C mammal). In recent literature, the hp, gp, pp are more commonly called the medial pallium (MP), dorsal pallium (DP), and lateral pallium (LP), reflecting their topology in the pallium of *Sarcopterygii*. Based on the absence or weak expression of the transcription factor *Emx1* within the LP, an additional subdivision, the ventral pallium (VP), was later added to the three classical ones (Fernandez *et al*. 1998; Puelles *et al*. 2000). However, the adult pallial area considered to the VP origin changes over time (Puelles *et al*. 2000, 2016; Medina *et al*. 2004), and some authors argue against the validity of this fourth pallial subdivision (Butler *et al*. 2011; Dugas‐Ford & Ragsdale 2015). Thus, for the sake of simplicity, we do not distinguish the LP and VP here.

The morphotype of the pallium of *Actinopterygii* was proposed mainly based on hodological (connectivity) and functional studies, and as mentioned above, the data were interpreted on the assumption that the medial‐lateral organization of the pallium in *Actinopterygii* is inverted from that in *Sarcopterygii*.

A set of studies in goldfish has suggested that a hippocampal‐like function (involved in spatial learning) resides in the lateral zone of the teleost pallium named Dl (Rodriguez *et al*. 2002), and an amygdaloid‐like function (involved in aversive learning) resides in the medial zone of the teleost pallium named Dm (Fig. 6C teleost; Portavella *et al*. 2004). Although a medio‐dorsal part of the pallium is also involved in spatial learning (Fig. 6C teleost, horizontal blue lines; Saito & Watanabe 2006), a simplified hypothesis “the Dl is hippocampal and the Dm is amygdaloid” has been widely accepted, because it fits better to the eversion theory (Northcutt 2006; Mueller *et al*. 2011). Other sets of studies in several teleost species have demonstrated that the sensory‐recipient areas are largely distributed throughout the pallium: visual inputs terminate in the Dl, auditory inputs in the Dm, and olfactory inputs in the Dp (Yamamoto *et al*. 2007; Northcutt 2008; Fig. 6C teleost).

If these functional organizations are homologous to those in mammals, as hypothesized in Figure 6A, we would expect that the hp‐like, gp‐like, pp‐like areas should originate from progenitor cell populations that are located from dorsal to ventral order within the early embryonic neural tube. Instead, based on the developmental study described above (Fig. 6B), the Dl containing visual‐recipient (gp‐like) and hippocampal‐like areas, and the Dp containing an olfactory (piriform‐like) area are all derived from the same set of progenitors located in the dorsal tip of the embryonic pallium (Fig. 6B,C teleost; blue areas). Most notably, the Dp is expected to originate from the ventral part of the pallium close to the subpallium, if it is regionally homologous to the piriform cortex in mammals (Fig. 6A; red areas). To account for the inconsistency, a hypothesis states that Dp cells migrate from the medial to the lateral part during the development (Mueller *et al*. 2011), but the cell lineage study clearly shows this is not the case. Thus, the current proposals of pallial subdivisions are questionable.

The existence of the three classical pallial subdivisions is ambiguous, which is also true for the amphibian pallium. The amphibian pallium has a simple tube‐like structure, which is subdivided into the medial, dorsal and lateral pallia, the same terminology applied to the vertebrate morphotype (the amphibian structures here will be abbreviated with lower‐case letters “mp, dp, and lp” to distinguish from the vertebrate morphotype subdivisions). In the time of Holmgren (1925), they were considered to have a one to one correspondence. Nonetheless, hodological studies have revealed that olfactory projections extend to dp, and “cortical‐like” projection patterns (inputs from the thalamus and distal output projections) are found more in mp than dp (Fig. 6C amphibian). Thus, the proposed hypothesis of homology is quite confusing: the mp in amphibians being homologous to the MP/DP in mammals, and the dp/lp in amphibians being homologous to the LP in mammals (Bruce and Braford 2009).

One could claim that the functional or hodological similarity should not be used as criteria for homology because they can change during the evolution. However, it should be noted that the definition of the classical three subdivisions, the hippocampal pallium, the general pallium, and the piriform pallium, reflects their functional properties. Other than the mammalian cortex (in which hippocampus and piriform cortices are three‐layered while neocortex is six‐layered), hodological and functional data have been the main criteria to identify the MP/DP/LP. Thus, if the use of functional or hodological properties to identify homology is questioned, we would need to doubt the presence of the MP/DP/LP subdivisions.

It may be worth reconsidering whether the MP (hp), DP (gp), and LP/VP (pp) are true subdivisions present throughout the vertebrates (Fig. 6D). Although the vertebrate pallia have several cytoarchitectonic subdivisions, they do not fit the hypothetical morphotype divisions in a simple one to one manner.

Identifying the neocortex homologue in non‐mammalian species has been a long‐lasting question, as it relates to the evolution of cognition. The area functionally similar to the mammalian neocortex receiving thalamo‐cortical‐like inputs (gp‐like) can be observed in different parts of the pallium of *Osteichthyes* (see slanted green lines in Fig. 6C), not necessarily in the presumable DP (central portion of the pallium as indicated in green in Fig. 4A). When observing the differential location of the gp‐like areas across vertebrates, one can think that any pallial region could actually evolve properties similar to the mammalian neocortex.

## Conclusion

Our comparative analyses suggest that the presence of the three regions (telencephalon, ORR, hypothalamus) in the secondary prosencephalon is conserved in vertebrates. However, the organization of each subregion is highly diversified. Current models of the brain organization is based on a mammalian‐centric view, but recent studies in teleost brains, in comparison with tetrapod brains, have allowed a better understanding of general organization of the brains of *Osteichthyes*. More data are needed for a concrete scenario of the regional homology of the vertebrate brain, and it is important to take into account a large range of species.
